# The Effect of Diagnostic and Therapeutic Changes on the Survival of Hodgkin’s Lymphoma Patients (1980–2019)

**DOI:** 10.3390/medicina60081272

**Published:** 2024-08-06

**Authors:** Árpád Illés, Boglárka Dobó, Fanni Borics, Dávid Tóthfalusi, László Imre Pinczés, Zsófia Miltényi

**Affiliations:** 1 Division of Hematology, Department of Internal Medicine, Faculty of Medicine, University of Debrecen, 4032 Debrecen, Hungary; dobo.boglarka@med.unideb.hu (B.D.); borics.fanni@med.unideb.hu (F.B.); tothfalusi.david@med.unideb.hu (D.T.); miltenyi.zsofia@med.unideb.hu (Z.M.); 2 Doctoral School of Clinical Medicine, University of Debrecen, 4032 Debrecen, Hungary

**Keywords:** Hodgkin lymphoma, PET/CT, survival, treatment

## Abstract

*Background and Objectives:* The overall- and progression-free survival rates of Hodgkin’s lymphoma patients have improved. Our goal was to examine the changes in our treatment results and their causes depending on the daily diagnostic and therapeutic practice. *Materials and Methods:* We analysed data of 776 classical Hodgkin lymphoma patients treated between 1980 and 2019. Patient data were investigated in ten-year periods (first period: 1980–1989, second period: 1990–1999, third period: 2000–2009, and fourth period: 2010–2019). *Results:* Radiotherapy alone as a first-line treatment was used progressively less often, and in the 4th period it was no longer used before or without chemotherapy. The use of combined chemo- and radiotherapy decreased in the last period, and the number of those patients who received only chemotherapy increased significantly. The 10-year overall survival improved significantly from 1990 to 1999 compared to 2010 to 2019 (74.9% vs. 86.9%). About 30% of patients relapsed after or were refractory to first-line therapy in each period. The incidence of relapse in the last period did not increase after two years, but there was no significant difference between the periods. *Conclusions:* Overall survival rates of HL patients have improved significantly in recent decades, which is due to improved diagnostic methods and modern therapies. Progression-free survival is unchanged; one-third of patients relapse or are refractory to first-line treatment within the first two years. Early recognition of R/R patients, the early application of newer and already available innovative therapies, and the finding of additional new and effective therapies are of particular importance.

## 1. Introduction

The survival of HL patients has improved continuously in the past decades, and today, 80–90% of them can be cured [1,2]. Diagnostic development and therapeutic changes play a significant role in this. The ^18^FDG-PET/CT (PET/CT) method is now the basis of staging at the time of diagnosis; furthermore, it is now routinely used several times during treatment, and the response-adapted treatment is based on it. First, with accurate staging at the time of diagnosis, it helps to avoid under- and overtreatment, plan the treatment precisely, and provide a basis for comparison for future examinations, thereby increasing their accuracy. An interim PET/CT during the treatment and a restaging examination after the end of the treatment are recommended, and based on the results, a modification of therapy is recommended if necessary. In Hungary, the basis of treatment between 1980 and 2000 was primarily radiotherapy, and, starting from the 2000s, ABVD polychemotherapy with irradiation was administered if necessary [3,4]. Since 2010, new, innovative drugs have also become available, such as brentuximab vedotin and immune checkpoint inhibitors, which can be used more frequently and are moving forward in the therapeutic line. The treatment strategy depends on the stage of the disease, the prognostic factors, and the patient’s age, general condition, and comorbidities, while also taking into account the available healthcare options in each country. Currently, combined treatment (ABVD + involved site radiotherapy) is generally recommended in the early stages, but this results in only about a 5% (+/−3%) progression-free survival advantage compared to those treated with chemotherapy alone. However, there is no difference in overall survival, thanks to the newer therapeutic options [5,6,7]. Based on clinical trials, it is possible to choose between combined treatment or chemotherapy alone based on the results of the PET/CT (interim) scan performed after the 2nd cycle of ABVD [8]. In advanced stages, if the interim PET/CT is negative after two cycles of ABVD, bleomycin can be omitted, and an additional four cycles of AVD treatment are recommended. Based on the results of the ECHELON-1 trial [9], the FDA and EMEA have approved the brentuximab vedotin–AVD (A-AVD) treatment for first-line therapy in advanced-stage HL patients [10]. BV is also available in Hungary as a first-line treatment for advanced-stage patients since 2020. Apart from BV, however, no new, innovative drugs are yet available for the first-line treatment of HL patients, but clinical trials are ongoing. However, the use of PET/CT and conventional but now PET-guided treatments has transformed our everyday practice.

Our goal was to examine these changes in our treatment results as well as their causes depending on the daily diagnostic and therapeutic practice.

## 2. Materials and Methods

The data of 776 classical HL patients, who were treated in Hungary at the Hematology Clinic of the University of Debrecen from 1 January 1980 until 31 December 2019 have been analysed. Patient data were investigated in ten-year periods (first period: 1980–1989, second period: 1990–1999, third period: 2000–2009, and fourth period: 2010–2019). Histological classification was always performed according to the currently valid classification/Rye’s classification [11], REAL/WHO classification [12], WHO 2008 classification [13], and WHO 2016 classification [14].

### 2.1. Staging and Response Assessment

Clinical staging was conducted according to the Ann Arbor Classification [15] in the first period, later with its Cotswold’s modification [16], and in the last period based on the Lugano classification [17]. Staging of the disease was based on 2-way chest X-ray, cervical, axillary, abdominal, and pelvic ultrasound, whole-body CT (neck, thoracic, abdominal, and pelvic), and a unilateral crista biopsy for the examination of the bone marrow in the first periods (1980–1989 and 1990–1999). Staging was based on CT scans in the 3rd period (2000–2009) and ^18^FDG-PET/CT examination—which became available from 2006—in the last period (2010–2019). Since then, ^18^FDG-PET/CT has been a standard staging procedure in HL.

Response assessment was performed according to Lugano classification and interpreted by the Deauville 5-point scoring system (DS). In the case of DS positivity, re-biopsy and treatment intensification may become necessary. A decision would be based on the clinical symptoms and changing of the parameters of the affected lymph nodes compared to the stage examination (SUVmax and the size of the affected lymph nodes). Patients’ therapy was based on the current therapeutic protocols (radiotherapy, chemotherapy, or combined radio- and chemotherapy).

### 2.2. Chemotherapy

The change in chemotherapy protocols was made between periods due to their effectiveness on the one hand and their side effect profile on the other. Primary polychemotherapy was dominantly CV(O)PP (cyclophosphamide, vinblastine (vincristine), procarbazine, and prednisone) in the first period, and ABVD (adriamycin, bleomycin, vinblastine, and dacarbazine) therapy is the basis of the treatment after 1999 (between 2000 and 2009 and 2010 and 2019). Salvage protocols were mainly BEACOPP (bleomycin, etoposide, adriamycin, cyclophosphamide, vincristine, procarbazine, and prednisone) and CEP (CCNU, etoposide, chlorambucil, and prednisone) and DHAP (dexamethasone, cytarabin, and cisplatin).

### 2.3. Radiotherapy

The irradiation initially consisted of extended-field, mantle-field, inverted Y, (sub)total nodal-or involved-field radiotherapy with a telecobalt machine until 1999.

### 2.4. Other Treatments

As a targeted immunotherapy, brentuximab vedotin (BV) was available in first-line treatment within clinical trials in the last period and through individual grant request use from 2020 onwards. Refractory disease (progression within 3 months after the end of the first-line treatment) and relapse (progression after 3 months after the first-line treatment) were treated with high-dose treatment and autologous hematopoietic stem cell transplantation (AHSCT) starting from the 1990s. In patients suitable for AHSCT, we use DHAP as the first salvage treatment, if we do not achieve remission with this, we use BV+bendamustin treatment, starting in 2016. After AHSCT, consolidation BV therapy has also been available as of 2016. For the treatment of refractory and relapsed (R/R) patients, we have used BV since 2016, and PD1 inhibitors in started being used in clinical trials in the last period.

### 2.5. Statistical Methods and Outcome Analysis

We analysed the changes in disease characteristics and overall- and progression-free survival in each period, also changes in survival outcomes in relation to treatment methods. Categorical variables are provided as their frequencies and percentages, while continuous variables are described with medians and ranges. Overall survival (OS) was defined as the time from diagnosis of HL to death from any cause. PFS was defined as the time from the date of HL diagnosis to disease progression, or death from any cause. Cox regression analysis was used to estimate hazard ratios (HRs) and respective 95% confidence intervals (CIs). Survival functions were calculated by using Kaplan–Meier estimates and comparison between categories was made using the log-rank test. The level of statistical significance was considered at *p* < 0.05. Statistical analyses were performed using SPSS 26.0 (IBM Corp., Armonk, NY, USA).

## 3. Results

We treated 776 patients with classical Hodgkin’s lymphoma between 1980 and 2019 (1980–1989: 192, 1990–1999: 199, 2000–2009: 226, and 2010–2019: 159 patients). Patient characteristics are listed in Table 1. The median follow-up time was 132 months (1–513).

### 3.1. Clinical and Treatment Characteristics

The male:female ratio decreased from period to period, then reversed in the last ten years. Most patients were diagnosed with an advanced disease stage in each era, except for era 3, when 52.65% of the patients had early-stage disease at the time of diagnosis. Between 2010 and 2019, significantly more patients were diagnosed with stage IV, other stages decreased compared to the previous period. The frequency of radiotherapy alone as a first-line treatment gradually decreased as the periods progressed until, in the 4th period, it was no longer used before or without chemotherapy. Combined chemo- and radiation therapy (CMT) became an increasingly common treatment modality until the 3rd period, then CMT decreased in the last period and the number of those patients who received only chemotherapy increased significantly (*p* < 0.001). About 30% of patients relapsed or were refractory (R/R) to first-line therapy in each period. Within R/R patients, the number of refractory patients increased during the periods (17%, 10%, 52%, and 69%).

### 3.2. Overall and Progression-Free Survival

**Figure 1:**
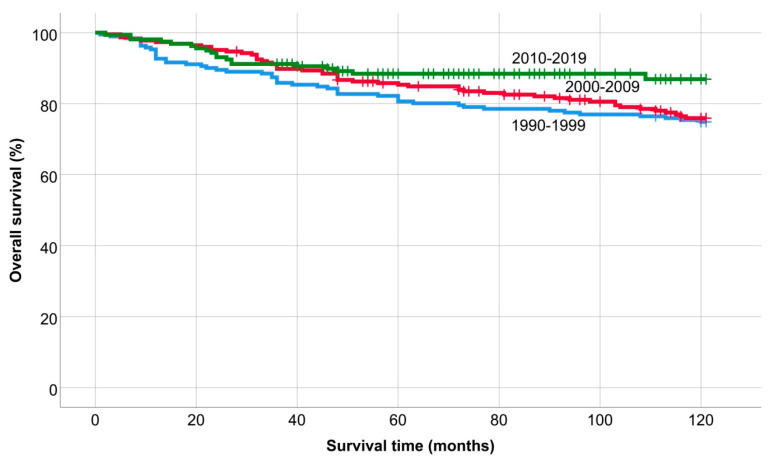
Overall survival of patients treated between 1990 and 1999, 2000 and 2009 and 2010 and 2019.

The overall- 5- and 10-year survival rates were 78.6% and 68.5% over the 40-year period. The exact time of the relapses was not known in patients who were treated between 1980 and 1989, so OS and PFS studies were not performed in this period. The overall 5- and 10-year survival of patients treated between 1990 and 2019 was 84.6% and 78%, and the 5- and 10-year progression-free survival was 65.2% and 58.9%. The 5-year OS was 80.6%, 85.3%, and 88.4%, and the 10-year OS was 74.9%, 75.9%, and 86.9% in each period (*p* = 0.06) (Figure 1). Overall survival improved significantly from 2010 to 2019 compared to 1990 to 1999 (*p* = 0.013).

**Figure 2:**
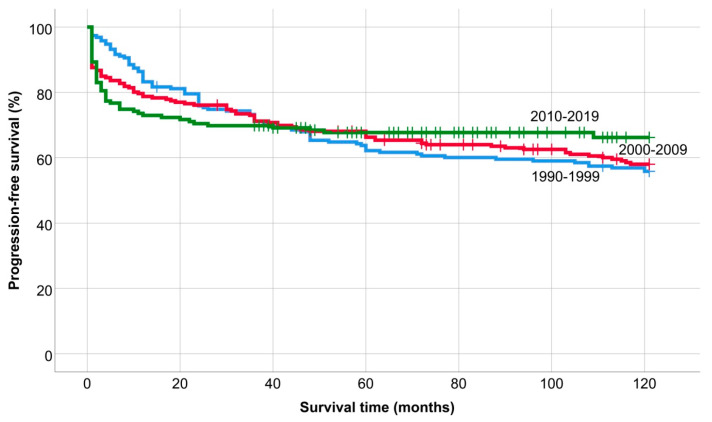
Progression-free survival of patients treated between 1990 and 1999, 2000 and 2009 and 2010 and 2019.

There was no significant change in the 5-year PFS, 62.2%, 66.3%, and 67.7%, and the 10-year PFS, 55.8%, 58% and 66.2% (*p* = 0.662), in the individual periods (Figure 2).

**Figure 3:**
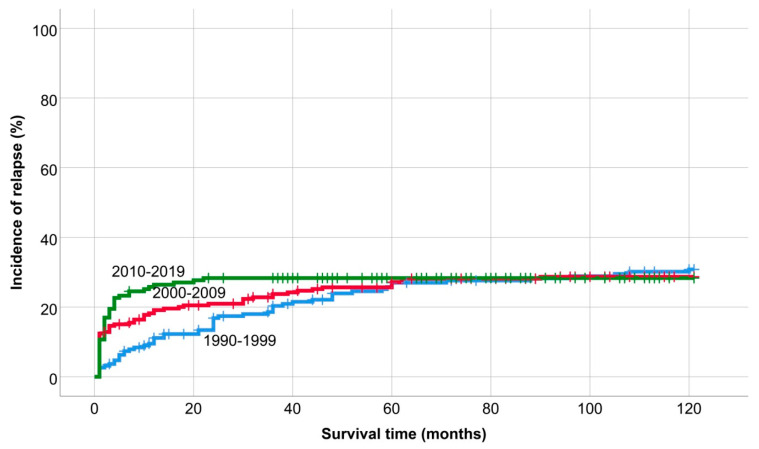
Incidence of relapse.

The incidence of relapse in the last period did not increase after two years, but there was no significant difference between the periods (Figure 3).

**Figure 4:**
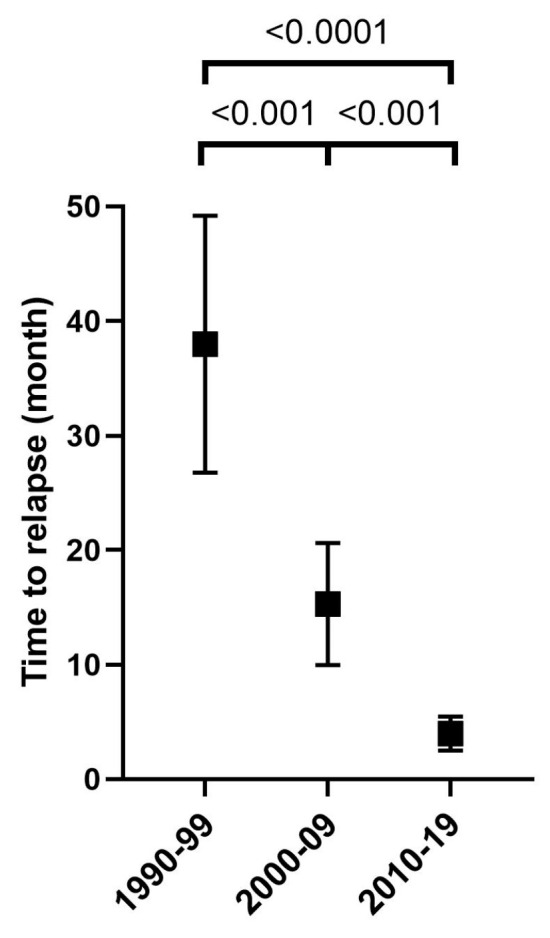
Time to relapse.

However, disease progression or relapse occurred significantly earlier over the course of the time periods (Figure 4).

**Figure 5:**
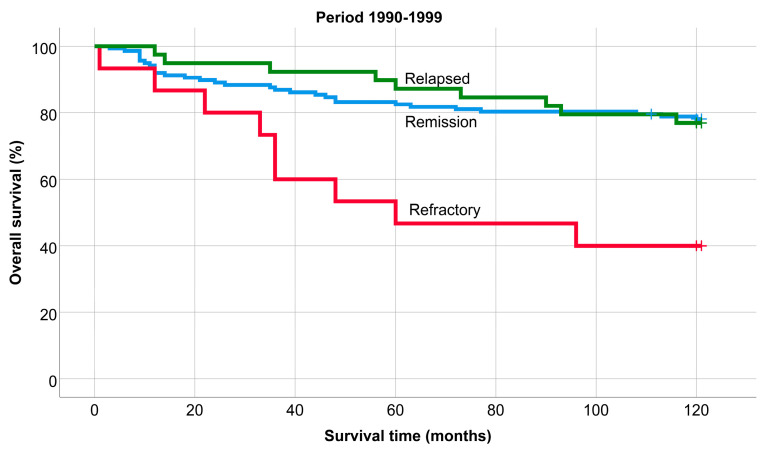
Overall survival of patients remaining in remission, relapsing, or being refractory, 1990–1999.

**Figure 6:**
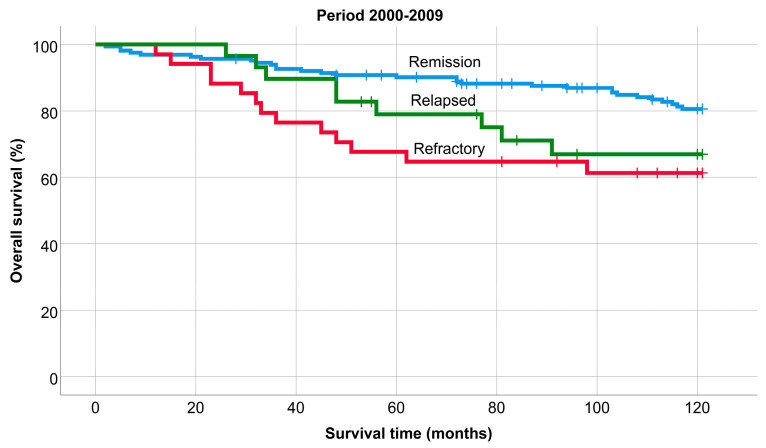
Overall survival of patients remaining in remission, relapsing, or being refractory, 2000–2009.

**Figure 7:**
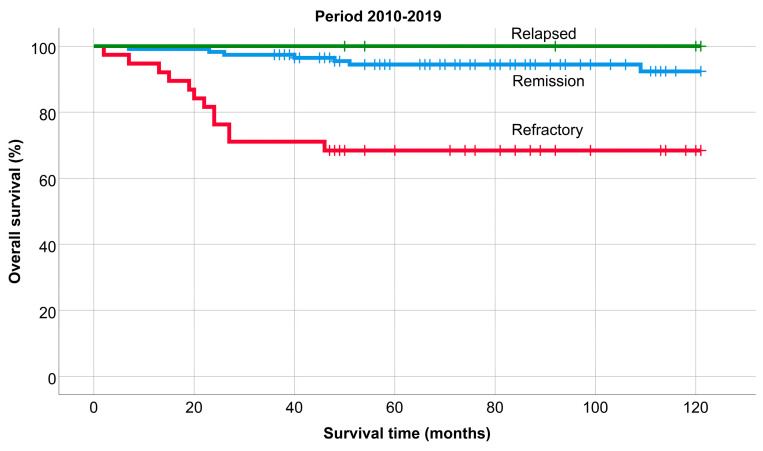
Overall survival of patients remaining in remission, relapsing or being refractory, 2010–2019.

The OS of patients remaining in remission, relapsing, or being refractory was significantly different in each period (*p* < 0.001); 5-year: 88.8%, 85.1%, and 64.1%, and 10-year: 82.5%, 74.8%, and 59.2%. (1990–1999: 78%, 77%, and 40% (*p*: 0.002), 2000–2009: 81%, 67%, and 61% (*p* = 0.01), and 2010–2019: 92%, 100%, and 68% (*p* < 0.001). Similarly, the survival of patients who remained in remission was always significantly better compared to refractory patients, while there was never a significant difference between the survival of patients who remained in remission and those who relapsed (Figure 5, Figure 6 and Figure 7).

In the last decade, the OS of patients who were in remission after first-line treatment and remained in remission was significantly better compared to the previous two periods (*p* = 0.002 and *p* = 0.026). The OS of refractory and relapsed patients did not change significantly; approximately 30% of refractory patients die (in the last period within the first two years). OS does not differ significantly according to stage (early vs. advanced) in any period. The 10-year PFS in the last two periods is significantly better in the early stage than in the advanced stage (69% vs. 52%).

## 4. Discussion

Until 2000, the first-line treatment for Hodgkin’s lymphoma in our centre was radiotherapy alone or with chemotherapy (COPP, then COPP/ABV). The treatment was significantly changed in 2000. Since 2000, the first-line chemotherapy has been ABVD and involved-field radiotherapy via a linear accelerator. Thanks to these modern treatments, our survival results have improved significantly.

### 4.1. Stage Migration

Compared to X-ray-, ultrasound- and CT-based approaches, PET/CT is much more accurate and sensitive in staging, resulting in a change in therapy in 3–25% of cases (upstaging in 9–41% and downstaging in 0–12%) [18]. The upstaging is mainly observed due to the detection of extranodal involvement (mostly bone marrow involvement) and the detection of lymph nodes with normal size (thus not diagnosed with CT), but with pathological activity. The reason for the downstaging is the normal uptake of larger lymph nodes (long diameter > 15 mm), which are considered pathological on CT [18]. Nowadays, it is performed at least three times during first-line treatment. First, for the staging, this helps to avoid undertreatment and overtreatment, to plan the treatment accurately, and provides a basis for comparison for subsequent examinations and thereby increases their accuracy. The interim PET/CT is performed after the 2nd cycle of the treatment, and the third (restaging) PET/CT is performed after the end of the treatment. These could help in the early recognition of refractory cases. Several criteria systems for the evaluation of the interim PET/CT were examined until, finally, the international validation study confirmed that the 5-point Deauville score (DS) is appropriate both in clinical examinations and in everyday practice [19]. We have used the DS since 2013 in everyday practice. A Deauville score of 1–3 is negative, while DS 4–5 is considered positive. In the case of negativity, it is recommended to continue the planned treatment. We observed an increase in the incidence of advanced-stage disease, especially stage IV, between 2010 and 2019. This is due to the routine use of PET/CT scans for more accurate diagnosis. Previous studies also confirmed the migration of the stages. Like our results, in Feinstein et al.’s study of adolescents and young adults, the number of patients diagnosed with stage I decreased significantly, while the proportion of patients with stage IV increased significantly, due to modern imaging techniques [20]. In contrast, Koshy et al. observed an increase in the proportion of stage II patients, but PET/CT was not yet routinely used in the examined period between 1983 and 2011 [21].

### 4.2. Change in Treatment Approach

During this period, MOPP (mechlorethamine, vincristine, procarbazine, and prednisone) treatment and its variants were mainly used, and 50–80% of patients with advanced stage HL achieved complete remission. Unfortunately, approximately one-third of them relapsed, and two late complications (sterility and a second hematologic malignancy) required replacement [22,23,24]. In our country, mustard nitrogen was not available, so we used the COPP protocol. In 1975, Bonadonna et al. described the ABVD (adriamycin, bleomycin, vinblastine, and dacarbazine) protocol, with which (as MOPP contains cytostatics that are not cross-resistant with ABVD) they successfully treated patients who did not respond to MOPP treatment or relapsed within a short time [25]. Recognizing the effectiveness of the treatment, it was also used as a first-line treatment alone or in combination with MOPP [23,26]. The combination was expected to reduce complications due to the different side effects and the lower doses used. Thus, COPP/ABV (cyclophosphamide, vincristine, procarbazine, prednisone/adriamycin, bleomycin, and vinblastine) was used as a first-line treatment in the second period. After that, it was confirmed that ABVD as a primary treatment is more effective than MOPP (CR: 82% vs. 67% and 5-year OS: 73% vs. 66%), and its side effect profile is also more favourable; moreover, compared to hybrid or alternative treatment/MOPP/ABV(D)/, their effects are equal and ABVD has fewer side effects [27]. ABVD (adriamycin, bleomycin, vinblastine, and dacarbazine) therapy has been the basis of treatment from 1999 (between 2000 and 2009 and 2010 and 2019). BEACOPP (bleomycin, etoposide, adriamycin, cyclophosphamide, vincristine, procarbazine, and prednisolone) treatment would be a possible option in the treatment of patients with advanced-stage disease; in our own practice, we do not use this because of its late complications (second malignancy and fertility problems) [28]. In a meta-analysis of seven chemotherapy protocols (MOPP, ABVD, MOPP + ABV, MOPP + ABVD, COPP + ABVD, Stanford V, and BEACOPP), the COPP + ABVD protocol proved to be the most effective therapy (best PR and ORR), but ABVD had the fewest side effects [29]. Supportive care has improved, enabling the use of ABVD since 2000, as the widespread routine prophylactic use of antiemetic treatments has made it possible to apply highly emetogenic chemotherapy. With the routinely used ABVD treatment today, late complications and the subsequent quality of life must focus on the application of adriamycin and bleomycin. Adriamycin, as an anthracycline drug, is cardiotoxic, and the pulmonary toxicity of bleomycin has long been known. An ECHELON-1 trial compared ABVD and brentuximab vedotin–AVD (A + AVD) treatment for first-line therapy in advanced-stage HL patients. The omission of bleomycin from the ABVD combination in these cases was necessary due to the increasing pulmonary toxicity [10]. During the use of A + AVD treatment, the most common side effect is polyneuropathy (86%), but 84% of these patients experienced complete resolution during follow-up. Primary prophylaxis with granulocyte colony-stimulating factor was recommended after an increased incidence of febrile neutropenia was observed with A + AVD (19.3% vs. 7.9%). The incidence was lower and similar to that observed with ABVD among patients who received A + AVD with G-CSF primary prophylaxis (10.8%). The occurrence of a second tumour was less common in the A + AVD group (3.5% vs. 4.9%) [10]. When using PD-1 inhibitor antibodies (nivolumab and pembrolizumab), their side effect profile is unique due to the enhancement of the immune response, leading to autoimmune phenomena (thyroiditis, pneumonitis, colitis, etc.). It has been recognized that the long-term survival of HL patients 10–15 years after treatment is no longer determined by the morbidity and mortality resulting from lymphoma itself, but by the late complications of the treatments [30]. Second malignancies and cardiovascular complications are the most common, and they are mainly related to radiotherapy [24]. A linear accelerator was installed in our centre in 2000, which made it possible to use the smaller radiation field; since then, we have used involved-field radiotherapy.

### 4.3. Changing of the Survival Results

#### 4.3.1. Overall Survival

As a result of the diagnostic and therapeutic changes, overall survival in the last period was significantly better compared to the 1990–1999 period. A significant OS improvement was observed only in the early stage of the last decade; there was no significant improvement in the advanced stage. This is similar to the results of Koshy et al., who also confirmed an improvement in the 5- and 15-year OS of patients diagnosed with early stage disease, while only an improvement of the 5-year OS was observed for advanced stages [21]. Recognizing the disease in its early stages can play a role in improving survival outcomes. Many studies examine the possibility of omitting radiation treatment in the early stages of HL. Based on the results so far, CMT has a PFS advantage of about 5% (+/−3%) compared to those treated with chemotherapy alone, but this overall survival advantage can no longer be proven thanks to the newer, innovative therapeutic options [5,6,7]. The use of radiotherapy has gradually decreased in our daily practice. If the risk of late side effects of radiation treatment is high (in the case of a family history of cumulative tumours, increased risk of breast tumour among young women with mediastinal involvement, increased risk of lymphoedema in the case of irradiation of the axillary region, increased risk of myocardial infarction in the case of coronary disease, etc.), we make an individualized decision in consultation with the patient about radiotherapy. However, in these cases, chemotherapy should be used in a larger number of cycles. In the case of an initial bulky tumour, we only recommend radiation treatment if PET positivity can be detected at the end of the treatment; because, in the case of PET negativity (both in the interim and in the restaging examination), the survival results are not significantly worse if the treatment is only with chemotherapy [31].

#### 4.3.2. Progression-Free Survival

There was no change in progression-free survival between the decades, even based on the stages (early vs. advanced). This may be due to the fact that we have used the same first-line treatments since 2000; although relapses are recognized earlier, they are in the same proportion. An improvement in the results of the first-line treatment is expected from the brentuximab vedotin treatment, and the FDA and the EMEA, based on the results of the ECHELON-1 study [9], also accepted the brentuximab vedotin–AVD (A + AVD) treatment for the first-line treatment of advanced-stage HL patients [9]. In Hungary, A + AVD treatment is currently available to stage IV patients with an individual fairness request. Escalated dose BEACOPP (bleomycin, etoposide, adriamycin, cyclophosphamide, vincristine, procarbazine, and prednisolone) treatment is a possible option in advanced-stage HL with a poor prognosis (IPS > 4, age < 60 years), but in our own practice, we do not use it due to late complications (second malignancy and fertility problems) [26]; in these cases, we use A + AVD therapy. According to the stages, the PFS in the last two periods proved to be significantly better in the early stage than in the advanced stage. Recognition of the disease in its early stages and appropriate treatment can improve the survival results. The proportion of R/R patients did not change significantly over the decades, but the number of refractory patients gradually increased compared to relapsed patients. As the time periods progressed, R/R cases were detected earlier in time. This is based on the use of restaging PET/CT (2–3 months after the end of the first-line treatment), which verifies the active disease sooner than with previous imaging tests. The improvement in OS is due in part to this early recognition and in part to better salvage therapies. This is also the explanation for the fact that the PFS of those treated between 2010 and 2019 is worse in the first two years, but no progression was observed after that. In contrast, in the other two periods, relapse was detectable even after two years. In summary, advances in active surveillance and diagnostic methods during follow-up allow for early detection of primary refractory and relapsing cases. Thanks to effective salvage treatments, there was never a significant difference between the survival of patients who remained in remission and those who relapsed. In the last decade, the OS of patients who remain in remission for first-line treatment has been significantly better compared to the previous two periods, and this may be due to less radiation treatments (and therefore less complications). The improvement of survival results was also confirmed in the study of Amzai et al. [32]. From the 1950s to 2013, the OS increased (from 30% to 86.6%). The survival results of 588 HL patients treated between 1980 and 2015 were examined. They used COPP and COPP-like chemotherapy before 2000 and ABVD treatment after 2000. Analysing and comparing the data of patients treated between 1980 and 2000 and 2005 and 2015, they found that the proportion of patients with an advanced-stage disease decreased from 61.1% to 48.1%. The 5- and 10-year OS were 61.3% and 51.8% in the first period, then 94% and 83.5%, which are like our results. The 5-year OS of R/R patients was 59.7% [32]. In our study, the OS of R/R patients improved, but not significantly, and we still lose approximately 30% of R/R patients. The OS of relapse patients is better than patients in complete remission in the first and last periods. This can be due to the smaller number of relapse patients, but it can also be attributed to a difference in the number of OS events resulting from other causes, independent of HL, and the fact that causes of death do not only involve the lymphoma. The investigation of causes of death has not been a purpose of this study. Considering that most HL patients are young, the treatment is basically curative. In R/R cases, autologous stem cell transplantation remains the standard treatment, supplemented with brentuximab vedotin treatment either before transplantation to achieve an adequate remission or after transplantation as consolidation therapy in patients at high risk of relapse. The survival results after the AHSCT are better if the patient is transplanted in complete metabolic remission (CMR), so it is absolutely necessary to try to achieve this. With traditional salvage treatments, complete remission can be achieved in 17–75% of cases [33]. If CMR is not achieved with traditional salvage treatments, brentuximab vedotin-based treatment is recommended. Although brentuximab vedotin alone showed a good response rate with a CR rate of 34% and an overall response rate (ORR) of 75% [34] in heavily pretreated patients, various BV-based combination therapies (BV-Bendamustine, BV-DHAP, BV-ESHAP, BV-gemcitabine, and BV-ICE) produce a much higher rate of CMR (69–81%), which is very important for the success of the transplant, and the ORR is also 74–95% [33]. For patients who do not reach CMR with these treatments, we use PD-1-blocking antibodies (nivolumab and pembrolizumab) before AHSCT, which enhance the immune response and therefore have a unique side effect profile. We must prepare for autoimmune phenomena (thyroiditis, pneumonitis, colitis, etc.). Currently, they are available with an individual fairness request before transplantation or in the case of relapse after transplantation and brentuximab vedotin treatment.

### 4.4. Predictors of Survival

According to univariate and multivariate analysis (Table 2 and Table 3), the OS was adversely affected by the presence of B symptoms and chemotherapy alone, while survival outcomes improved in patients treated in the last period. Additionally, the presence of B symptoms, advanced disease stage, and treatment with chemotherapy alone or radiotherapy alone were associated with a negative impact on PFS. However, assessing and comparing the impact of various treatments proved challenging and potentially misleading due to evolving treatment trends. Notably, the use of standalone RT gradually declined, with no such cases in the last decade. Similarly, the number of combination treatments also decreased. Although standalone RT posed a higher risk for shorter PFS compared to CMT, this distinction became less pronounced in OS. This shift was attributed to R/R patients essentially being treatment-naïve regarding chemotherapeutic agents. To address potential bias stemming from treatment differences, we conducted separate analyses for distinct time periods. The period of 1990–1999 underscored the predominant influence of classical prognostic parameters on OS. Consistent with this, the International Prognostic Score (IPS) by Hasenclever and Diehl, introduced in 1998 and still in use today, incorporates male sex as an independent prognostic factor, a finding we also confirmed. Over time, the prognostic significance of clinical parameters at diagnosis has diminished, aligning with advancements in diagnostic and therapeutic modalities. We attribute the decreasing impact of determinants on OS and PFS to the evolution of therapeutic options and the refinement of routine restaging assessments’ threshold limits, respectively. Figure 2 illustrates a comparable survival plateau for R/R patients, while early detection of suboptimal responders has expedited relapse identification. It is crucial to underscore that, in cHL, the choice of treatment modality and plan is dictated by the disease stage at diagnosis (number of chemotherapy cycles) and restaging assessment results (radiotherapy consolidation). Consequently, our multivariate analysis results cannot be directly used to compare therapeutic modalities. Instead, they may highlight specific patient groups with potential for treatment improvement, identifying the target population for future randomized controlled trials.

### 4.5. Future Therapies

In early stages, the main question today is whether to omit radiotherapy and define PET-guided therapies given the excellent OS and PFS results. The BREACH trial is the only randomized phase 2 trial incorporating BV in early-stage cHL. The study compared four cycles of BV-AVD + 30 Gy involved-node (IN)RT vs. four cycles of ABVD + 30 Gy INRT in early unfavourable patients without a PET-adapted strategy. The 2-year PFS was 97.3% and 92.6% (BV-AVD vs. ABVD), with a significantly reduced 2-year PFS in the interim PET-positive group treated with ABVD (71.6%) compared to BV-AVD (93.8%). The potential omission of RT was not examined [35]. The phase 3 RADAR trial compares ABVD vs. BV-AVD with interim PET-adjusted response and is ongoing. It will aim to answer the question of RT omission in early-stage patients [36].

The NIVAHL phase 2 randomized trial compared the concomitant administration of four cycles of nivolumab plus AVD (N-AVD) and a sequential treatment with four fortnightly infusions of nivolumab followed by two cycles of N-AVD and two cycles of AVD in early unfavourable patients. Three-year OS was 100% in both arms, and three-year PFS was 100% vs. 98% (concomitant vs. sequential treatment group) [37].

In advanced stages, improving PFS is the main goal, thus improving first-line therapy. Based on the results of the ECHELON-1 trial, A+AVD treatment is increasingly used for patients with advanced-stage cHL. The 6-year OS and PFS were better in the A + AVD group vs. ABVD group (93.9% and 89.4% and 82.3% vs. 74.5%). Fewer patients in the A + AVD group received subsequent therapy (20.4% vs. 23.8%) [38]. The randomized phase 3 trial, SWOG S1826, was conducted by the National Clinical Trials Network to evaluate nivolumab-AVD and BV-AVD in patients with advanced-stage HL. One-year PFS was 94% vs. 86% (N-AVD vs. BV-AVD). Fewer deaths occurred in the N-AVD group than with the BV-AVD group (4 vs. 11 deaths). Rates of febrile neutropenia were similar (5.6% vs. 6.4%), hypo/hyperthyroidism was more frequent after N-AVD (7%/3% vs. <1%), while sensory peripheral neuropathy was more common after BV-AVD (28.1% vs. 54.2%) [39]. The HD21, randomized, multicentre, parallel, open-label, phase 3 trial compared BrECADD and eBEACOPP treatment in advanced-stage HL. Treatment-related morbidity was significantly lower with BrECADD than eBEACOPP (42% vs. 59%). The estimated 4-year PFS was 94.3% vs. 90.9%, and the 4-year OS rates were 98.6% vs. 98.2%, respectively [40].

## 5. Conclusions

The overall survival of HL has improved significantly in recent decades, which is due to improved diagnostic methods and modern therapies. However, progression-free survival is unchanged; one-third of patients relapse or are refractory to first-line treatment within the first two years. Thus, the early recognition of R/R patients, the early application of newer and already available innovative therapies, and the finding of additional new, effective therapies are of particular importance.

## Figures and Tables

**Table 1:** Patients’ characteristics.

	1980–1989	1990–1999	2000–2009	2010–2019	
Patients	192	199	226	159	
Gender					
Male	108	109	115	77	*p* = 0.100
Female	84	90	111	82	
Stage					
I	11	9	22	5	*p* < 0.001
II	50	45	97	55	
III	97	96	52	24	
IV	27	32	51	75	
Early stages	61	54	119	60	*p* = 0.008
Advanced stages	124	128	104	99	
B signs	95	87	114	88	*p* = 0.134
Treatment					
radiotherapy alone	53	16	2	0	*p* < 0.001
chemotherapy alone	84	88	108	119	
combined chemo- and radiotherapy	23	77	115	40	
Follow-up					
alive	21	84	166	137	*p* < 0.001
death	170	114	59	21	
lost to follow-up	1	1	1	1	
Remain in remission	134	134	163	114	*p* < 0.001
Relapse	48	58	30	14	
Refractory (progression within 3 months after first-line treatment)	10	7	33	31	

**Table 2:** Univariate and multivariate regression analysis of all patients.

		All Patients			
		Univariate		Multivariate	
		HR (95% CI)	*p*	HR (95% CI)	*p*
OS					
Gender (vs. male)					
	Female	0.784 (0.545–1.129)	0.191		
Time of diagnosis (vs. 1990–1999)					
	2000–2009	0.910 (0.614–1.347)	0.637		
	2010–2019	0.536 (0.314–0.913)	0.022	0.405 (0.258–0.787)	0.005
Stage (vs. early)					
	Advanced	1.437 (0.979–2.110)	0.064		
B-symptoms (vs. absent)					
	Present	1.708 (1.182–2.466)	0.004	1.804 (1.222–2.662)	0.003
Treatment (vs. CMT)					
	RT	2.397 (0.932–6.164)	0.07		
	CT	2.125 (1.398–3.229)	<0.001	2.284 (1.496–3.488)	<0.001
PFS					
Gender (vs. male)					
	Female	0.893 (0.688–1.159)	0.395		
Time of diagnosis (vs. 1990–1999)					
	2000–2009	0.973 (0.724–1.306)	0.853		
	2010–2019	0.858 (0.607–1.213)	0.386		
Stage (vs. early)					
	Advanced	1.828 (1.375–2.432)	<0.001	1.592 (1.166–2.175)	0.003
B-symptoms (vs. absent)					
	Present	1.422 (1.096–1.846)	0.008	1.310 (0.986–1.739)	0.062
Treatment (vs. CMT)					
	RT	1.757 (1.317–2.345)	<0.001	2.968 (1.515–5.815)	0.003
	CT	2.427 (1.284–4.587)	0.006	1.602 (1.176–2.184)	0.002

**Table 3:** Univariate and multivariate regression analysis of patients stratified by the time period of diagnosis.

		1990–1999				2000–2009				2010–2019			
		Univariate		Multivariate		Univariate		Multivariate		Univariate		Multivariate	
		HR (95% CI)	*p*	HR (95% CI)	*p*	HR (95% CI)	*p*	HR (95% CI)	*p*	HR (95% CI)	*p*	HR (95% CI)	*p*
OS													
Gender (vs. male)													
	Female	0.526 (0.286–0.969)	0.039	0.556 (0.302–1.026)	0.06	1.039 (0.603–1.790)	0.891			1.066 (0.433–2.623)	0.890		
Stage (vs. early)													
	Advanced	0.949 (0.512–1.758)	0.868			1.643 (0.937–2.881)	0.083			2.511 (0.833–7.572)	0.102		
B-symptoms (vs. absent)													
	Present	2.342 (1.313–4.179)	0.004	2.254 (1.262–4.026)	0.006	1.349 (0.778–2.339)	0.286				0.216		
Treatment (vs. CMT)													
	RT	2.033 (0.732–5.647)	0.173			0 (0–>10)	0.976						
	CT	1.678 (0.863–3.261)	0.127			2.607 (1.443–4.712)	0.002			6.912 (0.919–51.987)	0.06		
PFS													
Gender (vs. male)													
	Female	0.718 (0.461–1.119)	0.143			0.962 (0.640–1.445)	0.850			1.104 (0.640–1.905)	0.722		
Stage (vs. early)													
	Advanced	1.139 (0.700–1.853)	0.601			2.501 (1.622–3.856)	<0.001	2.004 (1.244–3.227)	0.004	1.890 (1.024–3.491)	0.042	1.440 (0.756–2.744)	0.268
B-symptoms (vs. absent)													
	Present	1.670 (1.088–2.563)	0.019	1.977 (1.245–3.203)	0.004	1.564 (1.034–2.366)	0.034	1.302 (0.838–2.025)	0.241	1.055 (0.610–1.823)	0.849		
Treatment (vs. CMT)													
	RT	2.477 (1.231–4.983)	0.011	3.283 (1.577–6.833)	0.001	0 (0–>10)	0.965						
	CT	1.331 (0.817–2.170)	0.251			1.963 (1.288–2.992)	0.002	1.583 (1.003–2.497)	0.048	2.972 (1.267–6.970)	0.012	2.536 (1.035–6.214)	0.042

## Data Availability

Data sets generated during the current study are available from the corresponding author on reasonable request.
